# Molecular cloning and biochemical characterization of two cation chloride cotransporter subfamily members of *Hydra vulgaris*

**DOI:** 10.1371/journal.pone.0179968

**Published:** 2017-06-29

**Authors:** Anna-Maria Hartmann, Lucie I. Pisella, Igor Medina, Hans Gerd Nothwang

**Affiliations:** 1 Neurogenetics Group, School of Medicine and Health Sciences, Carl von Ossietzky University Oldenburg, Oldenburg, Germany; 2 Center for Neuroscience, Carl von Ossietzky University Oldenburg, Oldenburg, Germany; 3 INMED / INSERM Unité 901, Marseille, France; 4 Cluster of Excellence Hearing4All, Carl von Ossietzky University Oldenburg, Oldenburg, Germany; University of California Irvine, UNITED STATES

## Abstract

Cation Chloride Cotransporters (CCCs) comprise secondary active membrane proteins mainly mediating the symport of cations (Na^+^, K^+^) coupled with chloride (Cl^−^). They are divided into K^+^-Cl^−^ outward transporters (KCCs), the Na^+^-K^+^-Cl^−^ (NKCCs) and Na^+^-Cl^−^ (NCCs) inward transporters, the cation chloride cotransporter interacting protein CIP1, and the polyamine transporter CCC9. KCCs and N(K)CCs are established in the genome since eukaryotes and metazoans, respectively. Most of the physiological and functional data were obtained from vertebrate species. To get insights into the basal functional properties of KCCs and N(K)CCs in the metazoan lineage, we cloned and characterized KCC and N(K)CC from the cnidarian *Hydra vulgaris*. *Hv*KCC is composed of 1,032 amino-acid residues. Functional analyses revealed that *hv*KCC mediates a Na^+^-independent, Cl^−^ and K^+^ (Tl^+^)-dependent cotransport. The classification of *hv*KCC as a functional K-Cl cotransporter is furthermore supported by phylogenetic analyses and a similar structural organization. Interestingly, recently obtained physiological analyses indicate a role of cnidarian KCCs in hyposmotic volume regulation of nematocytes. *Hv*N(K)CC is composed of 965 amino-acid residues. Phylogenetic analyses and structural organization suggest that *hv*N(K)CC is a member of the N(K)CC subfamily. However, no inorganic ion cotransport function could be detected using different buffer conditions. Thus, *hv*N(K)CC is a N(K)CC subfamily member without a detectable inorganic ion cotransporter function. Taken together, the data identify two non-bilaterian solute carrier 12 (SLC12) gene family members, thereby paving the way for a better understanding of the evolutionary paths of this important cotransporter family.

## Introduction

The Cation Chloride Cotransporter (CCC) family consists of secondary active membrane proteins mainly mediating the symport of cations (Na^+^, K^+^) coupled with chloride (Cl^−^). Gene duplication events at the base of archaeans and eukaryotes caused their diversification into four subfamilies: the K^+^-Cl^−^ outward transporters (KCCs), the Na^+^-K^+^-Cl^−^ (NKCCs) and Na^+^-Cl^−^ (NCCs) inward transporters, the cation chloride cotransporter interacting protein CIP1, and the polyamine transporter CCC9 [1,2,3]. Subsequent taxon-specific losses of various CCC subfamilies resulted in differential distribution among taxa [1]. KCC genes have been established in the genome since eukaryotes and N(K)CC since metazoan [1].

KCCs and N(K)CCs differ in the composition of inorganic ion substrates, the direction of ion cotransport, and in their structural organization [2,4]. Proteins of both subfamilies contain 12 highly conserved transmembrane domains (TMs), which are involved in ion translocation [2,5,6,7,8]. The TMs are flanked by intracellularly located N- and C-termini. The structural difference among KCCs and N(K)CCs relates to the position of a large extracellular loop (LEL). The LEL of KCCs is situated between TM5 and TM6 and the LEL of N(K)CCs is located between TM7 and TM8 [2,4,7,9].

To date, most physiological studies on CCCs have been performed in vertebrates. The data obtained revealed that CCCs play a major role in various physiological processes such as regulation of cell volume, setting of the intracellular Cl^−^ concentration [Cl^−^]_i_ in neurons, directional ion transport across epithelial cells, and secretion of K^+^ [3,10,11,12,13]. Dysregulation of CCCs is associated with human disorders like Andermann’s syndrome, Gitelman`s syndrome and Bartter`s syndrome [2,14,15,16], epilepsy [17,18,19,20,21,22,23,24,25], neuropathic pain [26], spasticity [27], autism [24,28,29], brain trauma [30], schizophrenia [24], deafness [15,31], and Alzheimer disease [32].

Only few functional studies of CCCs are available for non-vertebrate species. The genome of *Drosophila melanogaster* comprises five putative CCCs (two N(K)CC, one KCC, one CIP1 and one CCC9 isoform) [1,33]. Functional analyses indicate that one of the *dm*N(K)CC isoforms (CG4537) and *dm*KCC (KCC-2) have pronounced Na-K-Cl and K-Cl cotransport like functions, respectively [33,34]. *Dm*N(K)CC is expressed in the nervous system and in the gut suggesting a role in ionic homeostasis (33), whereas *dm*KCC is expressed in hermaphrodite-specific motor neurons and is important for the inhibitory neurotransmission (34). *Caenorhabditis elegans* contains seven CCC isoforms (two N(K)CC, three KCCs, one CIP1 and one CCC9 isoform), whereby *ce*-KCC1 and *ce*-KCC2 have a K-Cl cotransporter like function [35,36,37]. The physiological role of *ce*-KCC2 is to coordinate the development of the inhibitory neurotransmission and the synapse maturation (36). Finally, a CCC was reported in *Arabidopsis thaliana* (*At*CCC). Interestingly, phylogenetical analyses and the predicted structural organization suggested that this *At*CCC encodes a K-Cl cotransporter, whereas functional analysis revealed a Na-K-Cl cotransporter like function [38]. Thus, functional analyses are required to classify phylogenetically and structurally predicted CCC family members as KCCs or N(K)CCs.

Little is known about the properties of KCCs and N(K)CCs in non-bilaterian species. This is, however, important with respect to the emergence of various physiological functions in evolution, such as inhibitory synaptic transmission [1]. We therefore set out to characterize CCCs from *Hydra sp*. This species contains five CCC isoforms (three N(K)CCs, one KCC and one CCC9) [1]. To gain insight into their functional properties, we first cloned KCC and N(K)CC of *Hydra vulgaris*. The encoded proteins were then characterized using different parameters: phylogenetical relationship, genomic and structural organization, and transporter function. These analyses provided us with important insights into basal functional properties of cnidarian CCCs.

## Materials and methods

### Hydra culture

*Hydra vulgaris* were originally obtained from Thomas C. G. Bosch (Zoologisches Institut, Universität Kiel) or Helbig Lebendkulturen (Prien, Germany). *Hydra vulgaris* were maintained in shallow dishes at room temperature in Volvic water. Twice a week, hydrae were fed with *Daphnia spp*. and the media was changed. For RNA isolation, approximately 0.07 g of hydra was stored at– 80°C until use.

### Cloning of hydra CCCs

Total RNA of 0.07 g *Hydra vulgaris* was extracted by the guanidine thiocyanate method and stored at -80°C. Quality and quantity of RNA samples were assessed by gel electrophoresis and optical density measurements. Reverse transcription of total RNA (2–7 μg) was performed using standard protocols with random hexanucleotides and oligodT primers using the Revert Aid first stand cDNA synthesis kit (Fermentas, Schwerte, Germany). For amplification of the 5’ end, the Mint-2 cDNA synthesis kit (Evrogen, Moscow, Russia) was used with a mixture of gene specific *hv*KCC and *hv*N(K)CC, random hexanucleotides and the PlugOligo3M primers. The open reading frames of *Hydra vulgaris* KCC and N(K)CC were amplified in five overlapping fragments, using semi-nested PCR and primers listed in Table 1. PCR was performed for 30 cycles, annealing was at 46–58°C for 45 s, and elongation was at 72°C for 1–2 min. PCR products were cloned into pGEM-T easy vector (Promega, Mannheim, Deutschland) using standard protocols. Ligations were transformed into XL1 blue cells (Stratagene, Germany), cultivated on ampicillin plates, and positive clones sequenced (LGC-Genomics, Berlin, Germany).

**Table 1:** Primer list for cloning of *hv*KCC and *hv*N(K)CC.

*hm*KCC_1072rev	TCCAGAGCGATTTGAACCAGC
*hm*KCC_1009rev	AGTAAATGATGTTGTGATATC
*hm*KCC_1909rev	TAACTGACTTGCAAGACTTAT
*hm*KCC_1825rev	TTGCGGTCTCCAATTCTTGGT
*hm*KCC_1657for	TTTGTTTCAAGCTGGTATTAT
*hm*KCC_1600for	CGTTATTATCATTGGAGTTTA
*hm*KCC_437rev	GCACCACCAAACTCTGGTCC
*hm*KCC_339rev	TGTGAGCATTGTACAACAACAGC
*hm*KCC_194rev	GGTAAACACCAGCAATTGTACCC
*hm*N(K)CC_1195rev	TTTCCTGTTGCATCTCGTAC
*hm*N(K)CC_1099rev	CGGAATAGCTTTTTGTGGATC
*hm*N(K)CC_2035rev	TCCACATATTAAAAGTCCATA
*hm*N(K)CC_1771for	ATGTTTTTAATCAGTTGGTGG
*hm*NK(K)CC_Stoprev	TTATGAATAAACGGTTAAAAC
*hm*NK(K)CC_1984rev	CATGCTAGGACGAGAGGAAGG
*hm*N(K)CC_1708for	CCATCTTATAAGTATTACAAC
*hm*N(K)CC_433for	ATGCTTAATATTTGGGGTGTT
*hm*N(K)CC_613rev	TCATATAGTATGCTCCACCCCTT
*hm*N(K)CC_674rev	TTAGCAAGTGCAAAGACTACTCC
*hm*N(K)CC_434rev	GCACAACACCCCAAATATTAAGCA
*hm*N(K)CC_484rev	GCTTCTAAAACACCTGATTGCCC
*hm*N(K)CC_388rev	ATCCATCCAAATTTGAAGACCGC

To characterize *hv*KCC and *hv*N(K)CC function in human kidney cells (HEK293 cells), the codon usages of *hv*KCC and *hv*N(K)CC were optimized for *Homo sapiens* by the gene optimization tool from Invitrogen (Gene Art Gene optimizer). In addition, an N-terminal HA-tag was added for better detection of the proteins. N-terminal tags like EGFP were shown to not interfere with CCC function [39,40,41]. Sequences were synthesized by GenScript (Nanjing, China) and cloned into the mammalian pCDNA3.1/Zeo^−^ expression vector. To generate stable cell lines, *hv*KCC and *hv*N(K)CC encoding constructs were cloned into the mammalian pEF5/FRT/V5-dest expression vector. These clones were stably transfected into HEK293-Flp cells using the Flp-In System (Invitrogen, Karlsruhe, Germany). As a control, we used a previously reported HEK293^KCC2^ cell line (60).

### Bioinformatic analyses

Exon-intron boundaries of *hv*KCC and *hv*N(K)CC were annotated by aligning the coding region of *hv*KCC (XP_012555566.1) and *hv*N(K)CC (KY646162) against the genomic sequence of *Hydra vulgaris* strain 105 (NW_004167712.1 and NW_004167444.1).

The human protein sequences of *hs*NKCC1 (NP_001037_1), *hs*NKCC2 (NP_001171761_1), *hs*NCC (NP_000330.2), *hs*KCC1 (NP_005063.1), *hs*KCC2 (NP_065759.1), *hs*KCC3 (NP_598408.1), *hs*KCC4 (NP_006589.2), *hs*CCC9 (NP_078904.3), *hs*CIP1 (NP_064631.2), and the *Hydra vulgaris* protein sequences of *hv*KCC (XP_012555566.1) and *hv*N(K)CC were used to generate a multiple sequence alignment by applying the default settings in MUSCLE [42] as implemented in SeaView v4.4.2 [43] and manually improved by eye thereafter. The phylogenetic tree of CCCs was constructed using maximum-likelihood with a bootstrap analysis of 1,000 replicates (PhyML). The final tree was edited using FigTree [44].

The secondary structures of *hv*KCC and *hv*N(K)CC were predicted using the TOPCONS program (http://topcons.cbr.su.se/) [45]. Putative N-glycosylation sites were predicted using the program GlycoEP (http://www.imtech.res.in/raghava/glycoep/submit.html).

### Immunocytochemistry

For immunocytochemistry, HEK293 cells were seeded on 0.1 mg/ml poly-L-lysine-coated (PLL) coverslips and incubated for 36 hrs. After fixation for 10 min with 4% paraformaldehyde in 0.2 M phosphate buffer and three washes in PBS, cells were incubated with blocking solution (0.3% Triton X-100, 3% bovine serum albumin, 10% goat serum in PBS) for 30 min. All steps were performed at room temperature. Primary antibody solution (mouse anti-N1/12, recognizing KCC2 (Biolegend, San Diego, USA, dilution 1:1,000) or mouse anti-HA.11 (Biolegend, San Diego, USA, dilution 1:250) [46] were added in blocking solution for 30 min. After three wash steps with PBS for 5 min, a secondary antibody, conjugated to a fluorescent probe (1:1000; Alexa Fluor 494 goat anti-mouse (Invitrogen), was added. After washing, cells were mounted onto glass slides with Vectashield Hard Set (Vector laboratories, Burlingame, CA). Photomicrographs were taken using a Laser scanning microscope (Leica TCS SP2).

### Cell surface biotinylation

Cell surface expression levels were assessed by surface biotinylation. For this purpose, 90–95% confluent 10 cm culture dishes of stably transfected HEK293 cell lines with *rn*KCC2, *hv*KCC and *hv*N(K)CC were treated with membrane-impermeant Sulfo-NHS-SS-Biotin (Thermo Fisher Scientific), according to the provided protocol. After several washes and cell lysis, biotinylated proteins were recovered by a NeutrAvidin agarose column. After three rounds of washes, biotinylated proteins were eluted in sample buffer. Aliquots of cell homogenates and eluates were collected and analysed by immunoblot analysis.

To quantify the amount of cell surfaced expressed KCCs, dilution series of each sample were loaded onto a 10% SDS-polyacrylamide gel system. After separation and electrotransfer onto PVDF membranes, membranes were incubated with rabbit anti-cKCC2 (dilution 1:5,000; [46] or mouse anti-HA.11 (Biolegend, Germany), dilution 1:1000). After incubation for 2 hrs at room temperature, membranes were washed four times with TBS-T (20 mM Tris, 150 mM NaCl, 1% Tween, pH 7.5) and the secondary antibody donkey anti-rabbit IgG-HRP or goat-anti-mouse IgG HRP (Santa Cruz Biotechnology, Heidelberg, Germany) applied for 1 hr. After washing, bound antibodies were detected using an enhanced chemiluminescence assay (GE Healthcare) and a LAS-3000 documentation system (Fujifilm, Düsseldorf). Quantification of bands was performed using the MultiGauge System V3.1 (Fujifilm). Cell lysates corresponding to the total protein amount were set to 100%. Only data with recovery values of 100 ± 20% were included in the analysis. Three biological and three technical replicas were performed for each experiment. Data are given as mean ± SD. Significant differences between the groups were analyzed by a Student’s *t*-test.

### Determination of K^+^-Cl^−^ and Na^+^-K^+^-Cl^−^ cotransport

Transport activity was determined by measuring Cl^−^-dependent uptake of non-radioactive Tl^+^ or radioactive ^86^Rb^+^ in HEK293 cells. Uptake-measurements were done as previously described [47,48]. Either stable Flp-In cell lines or transiently transfected cells were used. For transient transfection, 6 μl Turbofect (Fermentas, Schwerte, Germany), 150 μl Opti-MEM (Invitrogen, Karlsruhe, Germany) and 3 μg DNA of *mm*NKCC1 or *hv*N(K)CC were mixed and incubated for 20 min at room temperature prior transfection. 24 hours after transfection, HEK293 cells were plated in a black-walled 96 well culture dish (Greiner Bio-One; Frickenhausen, Germany) at a concentration of 100,000 cells/well.

For measurement of KCC transport activity, the medium was replaced by 80 μl of a Na^+^ free flux medium containing 100 mM N-methyl-D-glucamine-chloride, 5 mM KCl, 2 mM CaCl_2_, 0.8 mM MgSO_4_, 5 mM glucose, 5 mM HEPES (pH 7.4) with or without 2 μM FlouZin-2 AM dye (Invitrogen) plus 0.2% (wt/vol) Pluronic F-127 (Invitrogen) on the next day. After incubation for 48 min at room temperature, cells were washed 3 times with 80 μl preincubation buffer and incubated for 15 min with 80 μl preincubation buffer plus 0.1 mM ouabain to block Na^+^/K^+^ ATPases. Thereafter, the culture dish was inserted into a fluorometer (Fluoroskan Accent, Thermo Scientific, Bremen, Germany) and the wells were injected with 40 μl 5 x thallium stimulation buffer (12 mM Tl_2_SO_4_, 100 mM NMDG, 5 mM Hepes, 2 mM CaSO_4_, 0.8 mM MgSO_4_, 5 mM glucose, pH 7.4).

For measurement of NKCC transport activity, the medium was replaced by Cl^−^ low medium (135 mM Na-gluconate, 1 mM CaCl_2_, 1 mM MgCl_2_, 1 mM Na_2_So_4_, 15 mM HEPES, 5 mM Glucose, pH 7.4) with or without 2 μM FlouZin-2 AM dye (Invitrogen) plus 0.2% (wt/vol) Pluronic F-127 (Invitrogen) for 1 hr at 37°C. After incubation, cells were washed 3 times with 80 μl Cl^−^ low buffer and incubated for 15 min with 80 μl Cl^−^ low buffer plus 0.1 mM ouabain and with or without 10 μM bumetanide (selective blocker of NKCCs). Thereafter, the culture plate was inserted into a fluorometer (Fluoroskan Accent) and the wells were injected with 20 μl 12 mM Tl_2_SO_4_ and 20 μl 810 mM NaCl. Fluorescence was determined in a kinetic dependent manner (excitation: 485 nm, emission 538 nm, 1 frame in 5 sec in a 200 sec time span). Activity was calculated with the initial values of the slope of Tl^+^-stimulated fluorescence increase by using linear regression.

For radioactive flux measurements, cells were harvested 48 hours after transfection, transferred to PLL coated 6 well cultures dishes and incubated for 3 hrs. For N(K)CC measurements, cells were washed twice with preincubation buffer (135 mM NaCl, 2.5 mM KCl, 1 mM CaCl_2_, 1 mM MgCl_2_, 15 mM Na-HEPES, 5 mM glucose, pH 7.4) and incubated for 10 min at 37°C. Afterwards the medium was replaced by different Cl^−^ low media (see S5B Fig) and incubated for 1 hr at 37°C. The medium was then replaced by Cl^−^ low media plus 0.1 mM ouabain with or without 10 μM bumetanide for 10 min at room temperature. Finally, cells were incubated with uptake media (135 mM NaCl, 1mM KCl, 1 mM CaCl_2_, 15 mM HEPES, 0.1 mM ouabain, +/- 10 μM bumetanide, 2 μCi/ml ^86^Rb^+^, pH 7.4) for 7 min. Afterwards, cells were washed 3 times with 1 ml of ice-cold termination buffer (135 mM Na-gluconate, 7,5 mM NaCl, 15 mM HEPES, pH 7,4). Cells were subsequently lysed in 500 μl 0.25 M NaOH for 1 hr and neutralized with 250 μl pure acetic acid. ^86^Rb^+^ uptake was assayed by Cerenkov radiation, and the protein amount was determined by BCA (Thermo Fisher Scientific, Bonn, Germany).

For statistical analysis, data groups were compared using a Student’s *t*-test and p < 0.05 was considered as statistically significant.

### Measurement using Cl^−^-sensitive probe

#### Cell culture

For recordings of the fluorescence of the Cl^−^-sensitive probe, neuroblastoma N2a cells were transiently transfected with a mixture of three cDNA constructs encoding: i) ClopHensorN [49,50] ii) human α1 subunit of the glycine receptor-channel (GlyR) and iii) *hv*KCC, *rn*KCC2 (KCC2 of *rattus norvegicus*), or empty pcDNA3.1 (mock). For transfection, 300 μl Opti-MEM (Invitrogen), 7 μl Lipofectamine 2000, and 1.7 μg DNA were mixed and incubated for 15 min at room temperature. The proportion of constructs for transfection was as follows: 0.3 μg ChlopHensorN + 0.7 μg GlyR + 0.7 μg *hv*KCC or *rn*KCC2 or pCDNA3.1. In the meantime, N2a cells were harvested and plated in 35 mm dishes containing coverslips pre-coated with poly-ethylene-imine. The transfection mixture was then applied to the cell suspension. After 24 hrs of incubation, 1 μM strychnine, a selective GlyR blocker, was applied to the cell media. Cells were used for experiments 2–3 days after transfection.

#### Imaging setup and fluorescence recording procedure

The ratiometric chloride indicator ChlopHensorN is a chimeric protein composed of a tandem tomato and an EGFP_T203Y_ mutant harbouring an halogen-binding site that allows selective measurement of intracellular Cl^−^ at an excitation spectrum below 458 nm [49,50]. The tandem tomato fluorescence, excited at 594 is insensitive to Cl^−^ changes and can be used as reference signal [50]. In the present study, the ratiometric fluorescence of ChlopHensorN was measured using an epifluorescence imaging setup mounted on an inverted Olympus microscope (IX71, Olympus, Rungis, France) equipped with a FITC/CY3 Dualband ET Filterset (F56-023) and additional single-band excitation and emission filters included in two filter wheels (Lambda 10-B, Sutter Instruments Company, Novato, USA). The Cl^−^-sensitive fluorescence of ChlopHensorN (F_436_) was obtained using fluorophore excitation with 436/20 filter (F49-436) and 520/40 emission filter (F47-520). The fluorescence of Cl^−^-insensitive tandem tomato (F_577_) was obtained using 577/25 excitation filter (F49-577) and 641/75 emission filter (F37-641). The fluorescence signal was sampled at 0.05 Hz using a CoolSNAP*HQ* Monochrome CCD camera and Metamorph software (Roper Scientific SAS, Evry, France). Excitation lasted 60 and 20 ms for F_436_ and F_577_, respectively. All recordings were performed using a LUCPlanFLN 20x Objective, NA 0.45 (Olympus, Rungis, France) that allowed simultaneous recording of 30–50 transfected cells. The ratiometric signal (R_577/436_) reflecting changes of [Cl]_i_ was obtained after off line arithmetic division of F_577_ and F_436_ images.

6 mm coverslips with N2A cells, which were transfected with ChlopHensorN, GlyR and *hv*KCC or *rn*KCC2, were placed onto an inverted microscope and were perfused with different solutions using an experimental paradigm described previously [51]. The perfusion started with the physiologically relevant S1 solution (150 mM NaCl, 2.5 mM KCl, 2 mM CaCl_2_, 2 mM MgCl_2_, 10 mM HEPES, pH 7.4) + 10 μM bumetanide (selective blocker of NKCCs) + 50 μM glycine (agonist of GlyR), that was applied for 15 min. Thereafter, to determine the R_577/436_ corresponding to the minimal level of [Cl]_i_ (R_min_), cells were perfused with S2 solution (156 mM Na gluconate, 2 mM KCl, 1 mM CaCl_2_, 1 mM MgCl_2_, 2,5 mM HEPES, pH 7.4 + 30 μM bumetanide + 50 μM glycine) for 2 min. The opening of Cl^−^ permeable GlyRs in the presence of solution S2 containing low extracellular ([Cl^−^]_o_) (6 mM) and [K^+^]_o_ (2 mM) resulted in rapid decrease of [Cl^−^]_i_. Afterwards, cells were perfused with S3 solution (150 mM NaCl, 33 mM KCl, 2 mM CaCl_2_, 2 mM MgCl_2_, 2,5 mM HEPES, pH 7.4 + 30 μM bumetanide + 50 μM glycine) for 7 min to maximally load the cells with Cl^−^ through GlyRs and to determine R_max_ values of the ratiometric fluorescence in each individual cell. After overload with Cl^−^, cells were again perfused with S1 solution (containing 1 μM strychnine to block GlyRs) provoking an extrusion of Cl^−^ through *rn*KCC2 or *hv*KCC in comparison to mock transfected cells. The osmolarity of all solutions was adjusted to 310 mOsm. All experiments were performed at 24–25°C.

To normalize the results, the level of R_min_ for each individual cell was considered as “0” and R_max_ was set to “1”.

## Results

### Cloning of *Hydra vulgaris* KCC and N(K)CC

**Fig 1:**
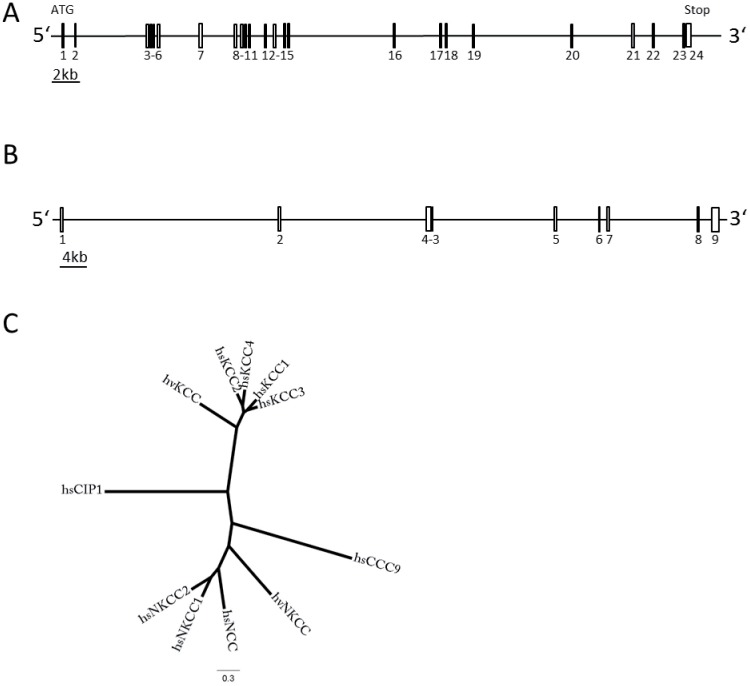
Gene organization and phylogenetic relationship of *Hydra vulgaris* KCC and N(K)CC. The gene organizations of *hv*KCC (A) and *hv*N(K)CC (B) were annotated using part of the genomic sequence of *Hydra vulgaris* strain 105 data derived from GenBank accession no. NW_004167712.1 and NW_004167444.1). Exons are symbolized by numbered boxes. Introns are horizontal lines between the exons, with lengths of line proportional to number of base pairs. (C) Phylogenetic tree of CCCs was constructed using the Maximum-Likelihood analysis (PhyML, model: Blosum62). The accession numbers are as follows: *hs*NKCC1 (NP_001037_1), *hs*NKCC2 (NP_001171761_1), *hs*NCC (NP_000330.2), *hs*KCC1 (NP_005063.1), *hs*KCC2 (NP_065759.1), *hs*KCC3 (NP_598408.1), *hs*KCC4 (NP_006589.2), *hs*CCC9 (NP_078904.3), *hs*CIP1 (NP_064631.2), *hv*KCC (XP_012555566.1), and *hv*N(K)CC (KY646162).

Recent phylogenetic analyses of CCCs revealed that KCCs and N(K)CC are present in non-bilaterian metazoans like cnidarians [1]. The genome of the cnidarian *Hydra magnipapillata* contains one KCC isoform and 3 N(K)CC isoforms [1]. To characterize their functional properties, the coding region of *Hydra vulgaris* KCC and N(K)CC, which is orthologous to the *Hydra magnipapillata* N(K)CC_isoform 1 (*hm*N(K)CC_isoform 1, XP_002159353.1), were cloned. The *hm*N(K)CC_isoform1 shows a higher average protein sequence identity to vertebrate N(K)CC isoforms than the *hm*N(K)CC_isoform 2 (XP_002170008_1, 32% instead of 21%) and a similar protein identity to *hm*N(K)CC_isoform 3 (XR_053545_1, 32%). To clone *hv*KCC and *hv*N(K)CC from *Hydra vulgaris*, gene-specific primers based on the nucleotide sequences of *hm*KCC and *hm*N(K)CC_isoform 1 were used. The 5’ and 3’ ends were obtained by rapid amplification of cDNA ends (RACE). The open reading frame of *Hydra vulgaris* KCC (*hv*KCC) encodes a 1,032 amino-acid protein (GenBank accession number: XP_012555566.1) and is composed of 24 exons that spans a total of 44.1 kb on the genomic level. Exon length varies from 51 bp to 206 bp and introns range from 88 bp to 7.3 kb (Fig 1A). The open reading frame of *hv*N(K)CC encodes a 965 amino-acid protein (GenBank accession number: KY646162). *Hv*N(K)CC comprises 9 exons within a genomic region of 87.9 kb. Exon length varies from 44 bp to 664 bp and introns range from 62 bp to 29.2 kb (Fig 1B).

To clarify the phylogenetical relation of *hv*KCC and *hv*N(K)CC to the different CCC subfamily members, a multi-sequence alignment of *hv*KCC, *hv*N(K)CC, and human CCC isoforms was generated and a phylogenetical tree constructed using the Maximum-Likelihood approach (S1 and S2 Figs). These analyses revealed that *hv*KCC clusters within the branch of human KCC isoforms (Fig 1C) and shares the highest amino-acid sequence identity with the human KCC1 isoform (48.1%). *Hv*N(K)CC is more closely related to human NKCCs and NCC isoforms (Fig 1C) and shares a sequence identity with the human NKCC2 isoform of 35.2%. Thus, we cloned for the first time non-bilaterian metazoan KCC and N(K)CC family members.

### Amino acid sequence analyses and topology of *hv*KCC and *hv*N(K)CC

**Fig 2:**
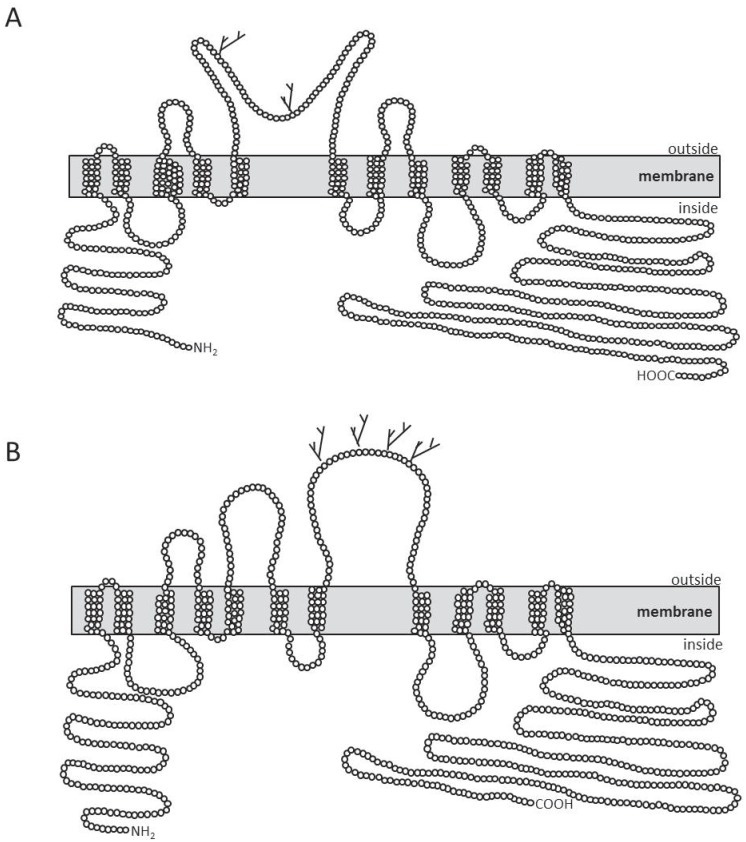
Two-dimensional structural organization of *Hydra vulgaris* KCC and N(K)CC. Putative topology of *hv*KCC (A) and *hv*N(K)CC (B) determined by using the TOPCONS program (http://topcons.cbr.su.se/). The grey box represents the membrane containing the 12 predicted TMs. Branched lines between TMs 5 + 6 indicate N-glycosylation sites which were predicted using the program GlycoEP (http://www.imtech.res.in/raghava/glycoep/submit.html).

Hydrophobicity profiles obtained from different prediction algorithms predict that CCCs consist of 10–13 transmembrane domains (TMs) flanked by large intracellular N- and C-termini [4]. Similar secondary structures were predicted for both *hv*KCC and *hv*N(K)CC using the web-based program TOPCONS. Most of the included prediction algorithms in TOPCONS indicate that *hv*KCC consists of 12–13 TMs with an LEL between TMs 5 + 6 (S3A Fig). The LEL was predicted to harbor two N-glycosylation sites. The separation between the last predicted TM (S1 Fig, dashed line) and the other TMs is greater than the separation between the other TMs. Comparison with the secondary structure of *hs*KCC1 revealed that this last TM was also observed for *hs*KCC1 by the TOPCONS program (S3B Fig). However, experimental data regarding several residues surrounding this putative TM clearly indicate that this sequence area is intracellularly located [52,53,54,55]. Due to the high conservation with the remaining human KCCs (71% sequence identity and 91% sequence similarity), we conclude that this TM is not a hydrophobic domain. Thus, we suggest a 2-dimensional model for *hv*KCC (Fig 2A), in which *hv*KCC consists of 12 TMs, intracellular termini, and an LEL between TMs 5 + 6 containing two N-glycosylated sites. The C-terminal domain harbors three highly conserved phosphorylation sites (*hv*KCC: Y887, T892 and Y1003 analogous to *rn*KCC2: Y903, T906 and Y1087), which are important for posttranslational regulation of mammalian KCCs [4,53,54,56,57] (S1 Fig).

Secondary structure analyses predicted for *hv*N(K)CC 11–12 TMs and an LEL between TMs 7 + 8. This is similar to the predicted 11–13 TMs and an LEL between TMs 7 + 8 for *hs*NKCC2 (S4 Fig). Experimental data demonstrated that N(K)CCs comprise 12 TMs and intracellular termini [9,58]. Based on the high conservation of TM domains (45.3% sequence identity to *hs*NKCC2), we propose a two-dimensional model in which *hv*N(K)CC comprises 12 TMs, intracellular termini, and an LEL between TMs 7 + 8 (Fig 2B). The LEL contains four predicted N-glycosylation sites (Fig 2B) and the N-terminus harbors two highly conserved Ser/Thr phosphorylation sites (*hv*N(K)CC: T61 analogous to *squalus acanthias* NKCC1: T184; *hv*N(K)CC: T84 analogous to *oryctolagus cuniculus* NKCC1: S126) [4,59]. Thus, the structural organization of *hv*KCC and *hv*N(K)CC is similar to the observed secondary structures of KCCs and N(K)CCs, respectively [2,4].

### Biochemical characterization of *hv*KCC and *hv*N(K)CC

**Fig 3:**
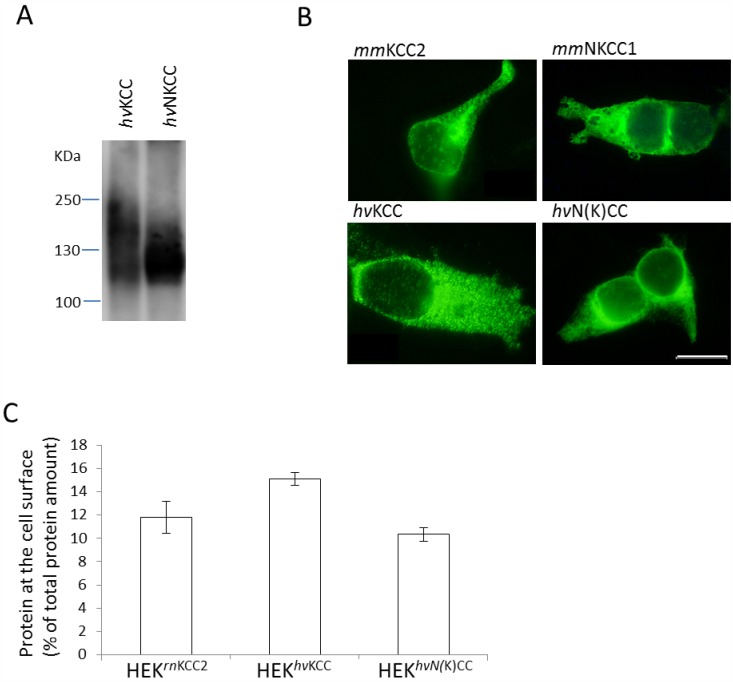
Biochemical characterization of *hv*KCC and *hv*N(K)CC. *Hv*KCC and *hv*N(K)CC were transiently (A, B) or stably transfected (C) in HEK293 cells. (A) Immunoblot analysis revealed a molecular mass of *hv*KCC and *hv*(N)KCC ranging from 115 to 215 kDa and 115 to 185 kDa, respectively. (B) Immunocytochemical analyses yielded subcellular distributions of *hv*KCC and *h*vNKCC similar to their mammalian counterparts (scale bar: 20 μm). (C) Cell surface expression analyses show that *hv*KCC (HEK^*hv*KCC^, 15.1 ± 0.5%) and *hv*N(K)CC (HEK^*hv*N(K)CC^, 10.3 ± 06%) are expressed at the cell membrane to a similar extent as *rn*KCC2 (HEK^*rn*KCC2^, 11.8 ± 1.4%).

Biochemical characterization of CCC family members included analyses of molecular masses, intracellular distribution, and transport activity properties. The calculated molecular mass of *hv*KCC and *hv*N(K)CC are 115 kDa and 108 kDa, respectively. To investigate the apparent molecular mass, HEK293 cells were transiently transfected with codon-optimized expression constructs for *hv*KCC and *hv*N(K)CC. Immunoblot analysis of detergent solubilized proteins detected *hv*KCC and *hv*N(K)CC as broad signals ranging from 115 to 215 kDa and 115 to 185 kDa, respectively (Fig 3A). The lower range fits well with the calculated molecular mass. The higher molecular weight bands likely reflect glycosylated, phosphorylated and dimeric forms as previously reported for mammalian CCCs [46,60,61,62,63,64].

The cellular distribution of *hv*KCC and *hv*N(K)CC in HEK293 cells was determined by immunocytochemistry. This analysis revealed a cellular distribution of *hv*KCC and *hv*N(K)CC similar to that observed for their mammalian mouse counterparts *mm*KCC2 and *mm*NKCC1 (Fig 3B). CCC-immunoreactivity was detected both at the plasma membrane and the perinuclear region. Only the nucleus was spared. An important prerequisite for their putative function as CCCs is the localization to the cell surface. To probe their presence in the cell membrane in a more precise way, biotinylation assays were performed using HEK293 cell lines stably expressing *hv*KCC, *hv*N(K)CC, or *rn*KCC2. Quantitative immunoblot analyses revealed that the percentages of plasma membrane bound *hv*KCC (HEK^*hv*KCC^, 15.1±0.5%) and *hv*N(K)CC (HEK^*hv*N(K)CC^, 10.3±0.6%) were similar to mammalian *rn*KCC2 (HEK^*rn*KCC2^, 11.8±1.4%) (Fig 3C).

In summary, these analyses demonstrate that the codon-optimized *hv*KCC and *hv*N(K)CC are well expressed in mammalian cells and that their cellular distribution is similar to mammalian CCCs. We therefore concluded that mammalian cells are suitable for functional characterization of *hv*KCC and *hv*N(K)CC.

### Functional analyses of *hv*KCC and *hv*N(K)CC

To investigate the functional properties of *hv*KCC and *hv*N(K)CC, fluorescence-based Tl^+^ and radioactive Rb^86^ influx measurements in HEK293 cells [47] and the chloride sensor efflux measurement method in neuroblastoma N2a cells [51] were used. To evaluate *hv*KCC for K-Cl cotransporter function, the following three criteria were applied: 1) measurement of K-Cl cotransport as a Cl^−^-dependent and Na^+^-independent transport of K^+^ ions in the influx and efflux operating mode, 2) inhibition of the cotransporter function by the CCC inhibitor furosemide, 3) stimulation of cotransport by N-ethylmaleimide (NEM) [37,65,66].

**Fig 4:**
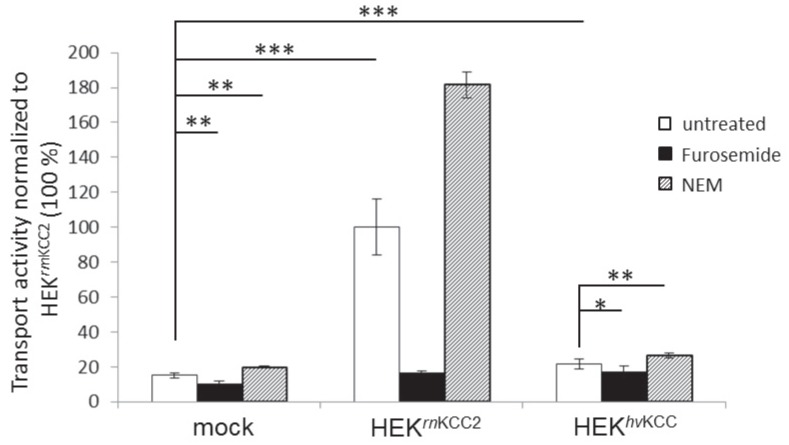
Thallium transport activity of *hv*KCC and mammalian KCC2. HEK293 were stably transfected with *rn*KCC2 or *hv*KCC. Thallium flux measurements revealed that *hv*KCC exhibits a Cl^−^-dependent, Na^+^-independent inwardly directed transport of K^+^ (Tl^+^ as a congener) that is 1.4 times higher than background. *:p < 0.05; **:p < 0.005; ***p < 0.001; n = 6.

Under physiological conditions, the K-Cl cotransporter has been considered to operate as a K^+^ and Cl^−^ dependent and Na^+^ independent net efflux cotransporter [65]. The direction of the net K-Cl cotransport is dependent on the affinities of the cotransporter for the ions and the sum of the K^+^ and Cl^−^ chemical gradients [65]. Thus, increasing the extracellular concentration of K^+^ (or as a congener Tl^+^) results in a net influx of K^+^ and Cl^−^ in several KCC isoforms [37,47,65,67]. This property is the basis for the Cl^−^-dependent, Na^+^-independent inward directed Tl^+^ based measurement technique. Tl^+^ based measurements in stably transfected HEK293 cells using a Na^+^ free flux medium revealed that *hv*KCC exhibits a transport activity (21.5 ± 2.8%, p = 0.00099) that is 1.4 times higher compared to the background (mock; 15.1 ± 1.4%) (Fig 4). Compared to other mammalian KCCs, *hv*KCC displayed a 4.8 times lower transport activity compared to *rn*KCC2 (100 ± 16.2%, p = 1.7 x 10^−8^, Fig 4). To further characterize the cotransporter properties, the impact of furosemide and NEM on the cotransporter function was investigated. Furosemide is a loop diuretic that inhibits the function of CCCs [66,68] and NEM is a cysteine-reactive compound that activates KCCs [69]. *hv*KCC can be significantly blocked by 2 mM furosemide (17.6 ± 2.9%, p = 0.04) and activated by 1 mM N-ethylmaleimide (NEM; 26.3 ± 1.5%, p = 0.003, Fig 4). Treatment of mock transfected cells (mock; 15.1 ± 1.4%) with furosemide (mock; 10.2 ± 1.5%, p = 0.002) or NEM (mock; 19.8 ± 0.7%, p = 0.005) resulted in a similarly significant inhibition or activation of endogenous HEK293 transporters. These similarities in the results for *hv*KCC and mock transfected cells make it difficult to determine whether NEM and furosemide influence *hv*KCC transporter function. However, our experiments reveal that *hv*KCC mediates a K^+^ dependent, Na^+^ independent influx similar to mammalian KCCs.

**Fig 5:**
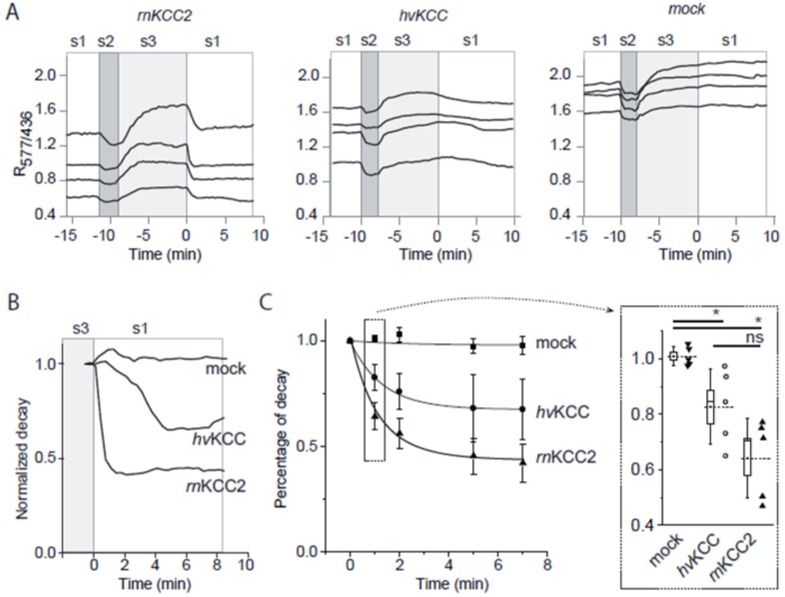
Cl^−^ extrusion in N2a cells cotransfected with ClopHensorN and *hv*KCC or *rn*KCC2. (A) Examplary records of R_577/436_ changes in individual cells expressing indicated constructs. Mock = pcDNA3.1. Zero time point is set at point of the initiation of K-Cl cotransporter-dependent Cl^−^ extrusion. (B) Examples of normalized mean traces of R_577/436_ decay recorded in individual experiment cells after transition from solution S3 (loading the cells with K^+^ and Cl^−^) to S1 (physiological buffer). 10 to 15 recorded cells per condition. (C) Pooled data from 5 experiments illustrating the mean ± SEM value of R_577/436_ at different time points. The inset illustrates box chart of results obtained 1 min after initiation of Cl^−^ extrusion, n = 5. Dotted lines indicate mean, boxes show SEM, the line inside box shows the median and the whiskers represent SD. Black arrowheads and blank circles indicate mean values from different experiments (10–15 cells per experiment and condition). *, p < 0.05; ns, non-significant, non-parametric Mann-Whitney test.

Next, we analyzed whether *hv*KCC operates in physiologically relevant conditions when the transporter uses the K^+^ gradient to extrude Cl^−^ from cells. To visualize [Cl^−^]_i_ changes, we used the Cl^−^-sensitive ratiometric probe ClopHensorN [49,50] that was transiently coexpressed in N2a cells together with GlyR and *hv*KCC, *rn*KCC2, or pcDNA3.1 (mock). The efficacy of K-Cl-dependent Cl^−^-extrusion was determined as decay of the Cl^−^-dependent component of R_577/436_ in cells overloaded with Cl^−^ through GlyR as described previously [51]. The transfer of the cell expressing *rn*KCC2 from Cl^−^-overloading solution S3 to physiologically relevant solution S1 resulted in rapid decrease of R_577/436_ fluorescence ratio in all transfected cells reflecting, presumably, the decrease of [Cl^−^]_i_ (Fig 5A and 5B). In contrast to *rn*KCC2 cells, the R_577/436_ signal in mock transfected cells remained stable for at least 10 min after cells transferred to the S1 solution (Fig 5A and 5B). This indicates the absence of an effective endogenous [Cl^−^]_i_ extrusion mechanism in N2a cells. The cells expressing *hv*KCC showed a weaker decay of R_577/436_ than *rn*KCC2, but the decay was clearly present (Fig 5A and 5B). Overall, the R_577/436_ decay curves in cells expressing *hv*KCC were significantly different from those measured in mock transfected cells (F(1,4) = 346.46; p = 4.9 x 10^−5^; two-way repeated measures ANOVA test, Fig 5C). The repeated R_577/436_ decay measurements in *hv*KCC were also significantly different from those in *rn*KCC2 (F(1,4) = 107.85; p = 4.85 x 10^−4^) indicating that the activity of *hv*KCC was lower than that of *rn*KCC2. This is in agreement with the Tl^+^ flux measurements. The statistical significance of the R_577/436_ difference between *hv*KCC and mock-transfected cells was further confirmed using the nonparametric Mann-Whitney test applied to the analysis of R_577/436_ decay 1 min after transfer to the solution S1 (Fig 5C, inset). Taken together, these results revealed that *hv*KCC mediates a Na^+^-independent, Cl^−^ and K^+^ (Tl^+^)-dependent cotransport in the efflux and influx operating modes.

**Fig 6:**
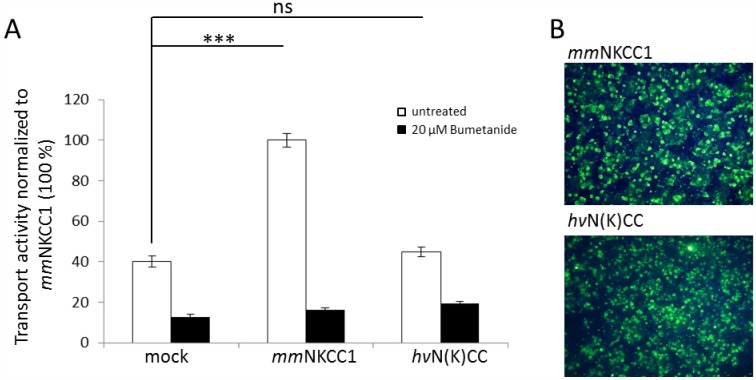
Thallium flux measurement of *mm*NKCC1 and *hv*N(K)CC. HEK293 cells were transiently transfected with *mm*NKCC1 and *hv*N(K)CC. (A) Thallium flux measurements according to [48] reveal that *hv*N(K)CC exhibits no significant transport activity compared to background (mock). (B) Immunocytochemical analyses show a similar expression rate of *mm*NKCC1 and *h*vN(K)CC, which were used for the transport activity measurements. *:p < 0.05; **:p < 0.005;***: p < 0.001; n = 3.

To test *hv*N(K)CC for Na-K-Cl cotransport, the following criteria are usually applied: 1) Na^+^, K^+^, Cl^−^ dependent influx, 2) inhibition of the cotransporter function by 10 μM of the NKCC specific inhibitor bumetanide, and 3) activation of transport activity by cell shrinkage [6,68,70,71,72]. The transport activity of *hv*N(K)CC was analyzed using the Na^+^, Cl^−^ dependent Tl^+^ or the Na^+^, Cl^−^ -dependent ^86^Rb^+^ based influx measurement methods [48,73]. In both cases, *hv*N(K)CC showed no significant transport activity compared to mock transfected cells (Fig 6, S5 Fig). The use of different preincubation buffer conditions (hypotonic, isotonic, hypertonic media, S5 Fig) in the ^86^Rb^+^ based influx measurement technique did not increase the transport activity of *hv*N(K)CC. In contrast, *mm*NKCC1 shows a 3.4 to 3.9-fold increased transport activity compared to the background in each of the tested conditions (Fig 6, S5 Fig). We concluded from these data that *hv*N(K)CC does not fulfill the criteria to be a functional Na-K-Cl dependent cotransporter when expressed in mammalian cells. However, we cannot exclude the possibility that *hv*N(K)CC is a functional Na-K-Cl cotransporter in a native environment.

In conclusion, functional analyzes revealed that *hv*KCC mediates a Na^+^-independent, Cl^−^ and K^+^ (Tl^+^)-dependent cotransport, whereas no inorganic ion-dependent transport activity was observed for *hv*N(K)CC.

## Discussion

CCC members are mainly involved in regulation of cell volume, setting of the intracellular Cl^−^ concentration [Cl^−^]_i_ in neurons, directional ion transport across epithelial cells, and K^+^ secretion. Their fundamental physiological importance is reflected in the fact that they have been established in the genome since the archaeans. Inorganic ion cotransporters subfamily members like KCCs and N(K)CCs first appeared in eukaryotes and metazoans, respectively. Here, we describe for the first time the functional characterization of KCCs and N(K)CCs from the non-bilaterian metazoan organism *Hydra vulgaris* (cnidarians).

Several properties are important in assigning a membrane protein to a specific family. These include the phylogenetic relationship, the genomic organization of the gene and the structural organization of the encoded protein, and similar functions [74,75,76,77]. Phylogenetical analyses of *hv*KCC, *hv*N(K)CC, and human CCCs established that *hv*KCC clusters within the branch of KCC subfamily members and therefore represents an orthologous KCC member. Previous phylogenetical analyses of cnidarian *Hydra magnipapillata* N(K)CCs suggest that there are three N(K)CC isoforms [1]. Here, we cloned and characterized the *hv*N(K)CC that is orthologous to the previously predicted *hm*N(K)CC_isoform1 (protein identity of 95%). Based on the phylogenetical analyses, *hv*N(K)CC is closely related to the N(K)CC subfamily and thus represents an orthologue of this CCC subfamily.

An intriguing feature of these proteins is their genomic organization. Vertebrate KCCs and N(K)CCs consist of 24–28 or 27–30 exons, respectively, with a minimal exon length of 45 bp [11,37,78,79,80]. The human KCC1 gene is the smallest gene, spanning 23 kb and the human NKCC1 is the longest one spanning 106 Kb [11,37]. The genomic organization of *hv*KCC with 24 exons, a minimal exon length of 54 bp and 44 kb in total length is very similar to its vertebrate counterparts. In contrast, *hv*N(K)CC comprises 9 exons with exon length ranging from 44 to 664 bp in a total span of 88 kb. Comparison with mouse NKCC1 (*mm*NKCC1) that exhibits 27 exons over a total length genomic length of 75 kb reveals that the average exon length of mouse NKCC1 (~110 bp) is 2.5 times lower than the average exon length of *hv*N(K)CC (~270 bp) [81]. Thus, *hv*N(K)CC comprises 9 exons, that are on average larger than their vertebrate counterparts. A similar observation was made for the genomic organization of the invertebrate *ce*-KCC1 gene which comprises 10 exons that are in average larger than the exon length of human KCC1 [37].

On the structural level, CCCs consist of a central, hydrophobic domain that is predicted to contain 12 TMs and intracellularly localized termini [2,4]. A major structural difference between KCCs and N(K)CCs is the localization of the LEL. KCCs have the LEL between TMs 5 and 6 [37,65,66,82,83] and N(K)CCs between TMs 7 and 8 [6,9,58,70,84,85]. Hydropathy plots and sequence similarities of the highly-conserved TMs among KCCs and N(K)CCs revealed that *hv*KCC and *hv*N(K)CC consist of 12 TMs and intracellular termini. The LEL of *hv*KCC and *hv*N(K)CC is situated between TMs 5 and 6 or TMs 7 and 8, respectively. Thus, phylogenetical and structural data concur to the conclusion that *hv*KCC is an orthologous subfamily member of KCCs and that *hv*N(K)CC belongs to the N(K)CC subfamily.

After heterologous expression of *hv*KCC in human HEK293 and N2A cells, *hv*KCC mediated a Na^+^-independent, Cl^−^-dependent cotransport of K^+^ (Tl^+^) ions in the influx and efflux operating mode. In combination with the phylogenetical relationship to the KCC subfamily, the similar genomic and structural organization, we conclude that *hv*KCC encodes a K-Cl cotransporter. Interestingly, recent investigations revealed that KCCs have been implicated in volume regulation in the anthozoan *Aiptasia diaphana* and may also regulate the hyposmotically-induced volume regulation of nematocytes [86]. Nematocytes are specialized secretory and sensorial cells of cnidarians which are used for predation and aggression strategies. Physiological analyses of isolated nematocytes demonstrated that a putative KCC mediated the Cl^−^ dependent K^+^ efflux which is important for volume regulation [86]. An important future task will be to analyze whether the cloned and characterized *hv*KCC described here is important for the volume regulation in hydra.

Phylogenetic analyses and the prediction structural organization suggest that *hv*N(K)CC is a member of the N(K)CC subfamily. However, no inorganic ion cotransport activity of *hv*N(K)CC was detected using different approaches. This is in agreement with the functional analyses of CCC subfamily members from *Drosophila melanogaster*. The genome of this fly contains two paralogous N(K)CC genes [1,33]. One of them exhibits a pronounced transport activity (CG4537), whereas the other isoform (CG31547) is transport inactive [33]. Thus, there is a possibility that one or both of the two other *hv*N(K)CC isoforms will display a Na-K-Cl cotransporter like function.

In conclusion, we have cloned and characterized the phylogenetical relationship, genomic and structural organization and the functional properties of KCC and N(K)CC from *Hydra vulgaris*. These analyses indicate that *hv*KCC encodes a functionally active K-Cl cotransporter, which probably has a physiological role in volume regulation of nematocytes. Furthermore, comparative analyses of the structure-functional relationship of functionally active K-Cl cotransporters in the metazoan lineage will provide profound insights into the precise ion translocation mechanism. The TMs, which are important for ion translocation, have a sequence identity to human KCCs of around 70%. Construction of chimeric proteins and mutagenesis studies of different TMs can provide profound information about which structural domains are important for the binding and transport of K^+^ and Cl^−^.

## Supporting information

S1 Fig Multi-alignment of *hv*KCC and the human KCC protein sequences. The multiple sequence alignment of *hv*KCC and human KCCs 1–4 and were generated using ClustalW. Transmembrane domains were predicted using the web-based program TOPCONs and marked with bars. Potential N-glycosylation sites are marked by blue circles (GlycoEP) and conserved phosphorylation sites are marked by asterisks. *hs*: *homo sapiens*, *hv*: *hydra vulgaris*.
(EPS)

S2 Fig Multi-alignment of *hv*N(K)CC and the protein sequences of human NKCC1, NKCC2 and NCC. The multiple sequence alignment of human *hv*N(K)CC, NKCC1, NKCC2 and NCCC was generated using ClustalW. Transmembrane domains were predicted using the web-based program TOPCONs and marked with bars. Potential N-glycosylation sites are marked by blue circles (GlycoEP). *hs*: *homo sapiens*, *hv*: *hydra vulgaris*.
(EPS)

S3 Fig TOPCONS analysis of the predicted transmembrane domains of *hv*KCC and *hs*KCC1. The topology of the transmembrane domains of *hv*KCC (A) and *hs*KCC1 (B) was predicted using the TOPCONS program (http://topcons.cbr.su.se/).
(EPS)

S4 Fig TOPCONS analysis of the predicted transmembrane domains of *hvN(*K)CC and *hs*NKCC2. The topology of the transmembrane domains of *hvN*(K)CC (A) and *hs*NKCC2 (B) was predicted using the TOPCONS program (http://topcons.cbr.su.se/).
(EPS)

S5 Fig Rubidium flux measurement of *mm*NKCC1 and *hv*N(K)CC. The transport activity of transiently transfected *mm*NKCC1 and *hv*N(K)CC were measured using the radioactive rubidium flux measurement method (A; n = 4). For this analysis, different buffer conditions were chosen (C). The rubidium flux measurement method revealed no significant transport activity of *hv*N(K)CC in any chosen buffer condition. (B) Immunocytochemical analyses were performed in parallel to the flux to verify similar transfection rates for the transport activity measurements.
(EPS)
